# The TNF receptor-ligands 4-1BB-4-1BBL and GITR-GITRL in NK cell responses

**DOI:** 10.3389/fimmu.2012.00402

**Published:** 2013-01-04

**Authors:** Isabel Barao

**Affiliations:** Department of Microbiology and Immunology, University of Nevada, RenoReno, NV, USA

**Keywords:** NK, leukemia, 4-1BB, GITR, therapy

## Abstract

Interactions between several tumor necrosis factor (TNF)-TNF receptor (TNFR) superfamily members that are expressed by T cells and natural killer (NK) cells and various other cell types modulate immune responses. This review summarizes the current understanding of how the TNF ligand-TNFR interactions 4-1BBL with 4-1BB, and GITRL with glucocorticoid-induced TNFR-related (GITR) regulate NK cell mediated antitumor responses and discuss its therapeutic implications.

## Natural killer cells

**Figure 1:**
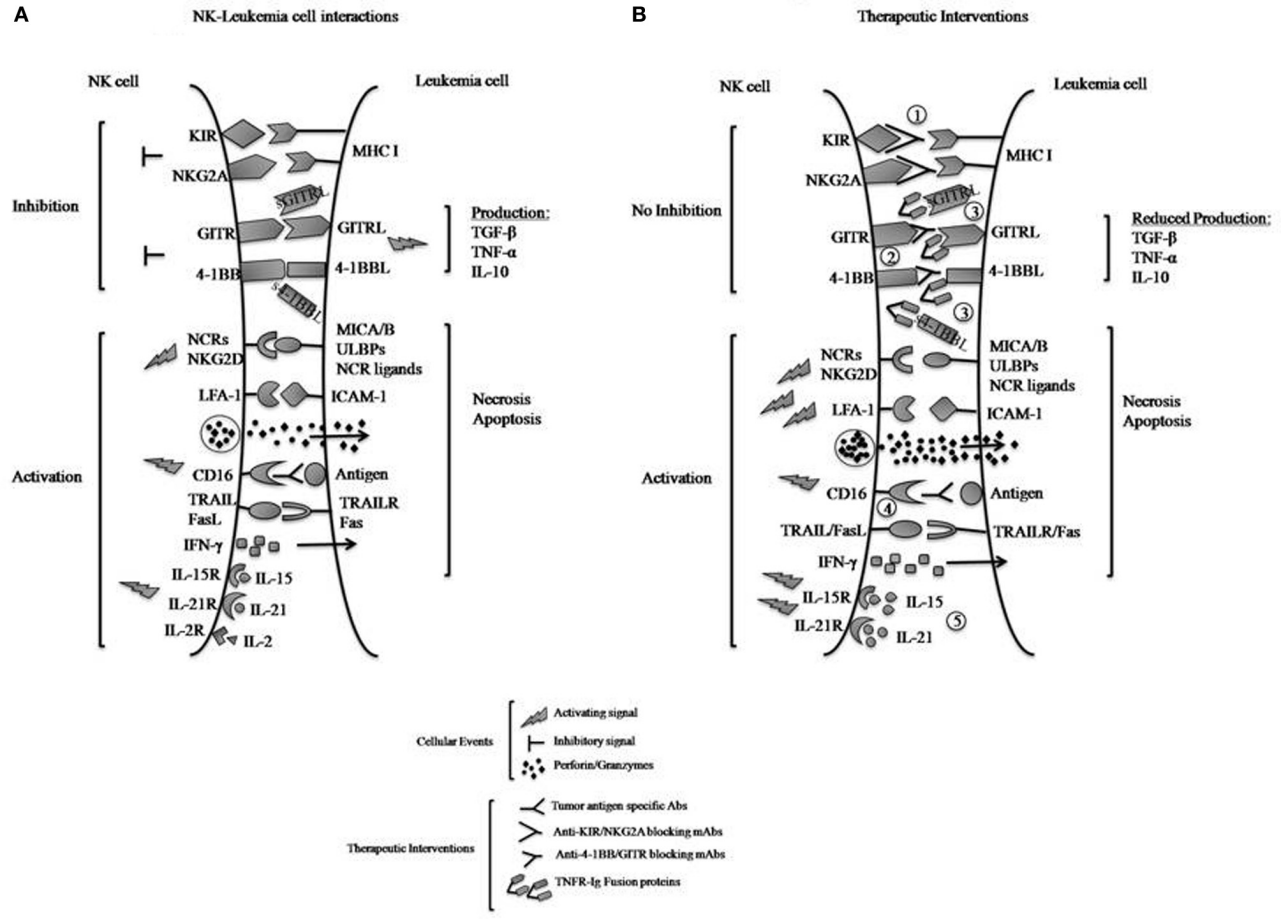
**(A)** Interactions of inhibitory and activating NK cell receptors, including TNF family receptors 4-1BB and GITR, with ligands expressed on leukemia cells. Engagement of the inhibitory receptors KIR and NKG2A with MHC class I molecules directly transmits inhibitory signals to the NK cells to reduce killing of tumors and IFN-γ production, whereas interactions of 4-1BB with 4-1BBL and GITR with GITRL can produce activating or inhibitory effects depending on conditions. Here their exclusively inhibitory effects upon interaction with leukemia cells are illustrated. Reverse signaling (by the bound ligands 4-1BBL and GITRL) to signal the leukemia cells induces the leukemic production of immunosuppressive cytokines such as TGF-β, TNF-α, and IL-10, which also suppress NK cell functions. Engagement of the activating NK cell receptors (NCRs) to their respective ligands and NKG2D to MICA/B or ULBPs on tumors transmits activating signals to NK cells and triggers their functions. Interactions between LFA-1 and ICAM-1 on the tumors promote cell-cell adhesion and activate NK cells. NK cell recognition of antibody-coated tumor cells by CD16 results in NK cell activation and tumor killing (ADCC). NK cells respond to cytokines such as IL-2, -15, or -21 by proliferating and increasing their functions. Activated NK cells can release lytic molecules such as perforin and granzymes upon engagement of target cells leading to necrosis and/or apoptosis of tumors. NK cells can also express TNF family proteins TRAIL and FasL, which binds death domain-containing TRAILR and Fas on tumors cells to induce tumor-cell apoptosis. **(B)** Therapeutic approaches to increase NK cell anti-leukemia effects. NK cell-mediated anti-leukemia activity can be enhanced by several approaches, including: (1) inactivation of KIR- and NKG2A-derived inhibitory signals using blocking mAbs; (2) blocking 4-1BB and GITR inhibitory signals using specific mAbs; (3) neutralization of 4-1BBL and GITRL effects on leukemia by competition with 4-1BB-Ig and GITR-Ig fusion proteins; and (4) ADCC with mAbs specific for tumor-associated antigens; and (5) IL-15 or IL-21 stimulation of cytotoxic activity. It should be noted that with approach 3, the R-Ig reagents may be able to stimulate the tumor productive of TGF-β and other immunosuppressive cytokines.

Natural killer (NK) cells are lymphocytes of the innate immune system that kill a variety of tumors and infected cells (by bacteria, parasites, and viruses) without prior sensitization to antigen and secrete cytokines that shape adaptive immune responses (Herberman et al., 1975; Kiessling et al., 1975; Vivier et al., 2008). Differing from T cells, NK cells lack antigen-specific receptors and bear a variety of cell surface activating and inhibitory receptors to recognize the cells that they kill and to regulate their functions (Figure 1A) (Lanier, 1998; Moretta et al., 2006; Baessler et al., 2010; Placke et al., 2010). In this review I will focus on how to exploit the inhibitory activity of tumor necrosis factor (TNF) receptors, 4-1BB and glucocorticoid-induced TNFR-related (GITR) in NK anti-leukemia activity to improve therapeutic NK cell intervention.

Major histocompatibility complex (MHC) class I-specific inhibitory receptors are particularly important for NK cells to discriminate “self” (healthy cells) from “altered self” (infected- and transformed-cells) or “missing self” (Karre et al., 1986; Bix et al., 1991). The MHC class I-specific inhibitory receptors include the killer cell immunoglobulin-like receptors (KIRs) in humans, the lectin-like Ly49 dimers in the mouse and the lectin-like CD94-NKG2A heterodimers in both species (Vilches and Parham, 2002; Yokoyama and Plougastel, 2003). By interacting with their respective MHC class I molecules [human leukocyte antigen (HLA) in humans and H-2 and Qa1, in mice], these receptors deliver inhibitory signals to the NK cell and prevent killing of healthy cells (tolerance to self). In contrast, down-regulation or loss of MHC class I expression (e.g., in microbe-infected cells, stressed or injured cells, and tumors) shifts the balance toward activation and renders target cells susceptible to NK cell-mediated lysis (Vilches and Parham, 2002) (Diefenbach and Raulet, 2001; Lanier, 2005). The NK cells may also express leukocyte immunoglobulin like receptors (LILR), some of which also recognize MHC class I ligands and deliver inhibitory signals upon engagement. There are many activating receptors, including the natural cytotoxicity receptors (NCRs; NKp30, NK40, NKp46), NKG2D, 2B4/CD244, CD2, LFA-1, co-receptors such as DNAM-1, and the Fc receptor CD16 (FcγRIIIa) that supports antibody-dependent cell-mediate cytotoxicity (ADCC) that is involved in many monoclonal antibody (mAb) anti-tumor therapies (Bottino et al., 2006; Schleinitz et al., 2008; Pegram et al., 2011). NK cells are activated by various stimuli such as contact with dendritic cells (DC), MHC-I-negative cells, binding of IgG-containing immune complexes, direct engagement of NK receptors by stress-induced tumor-associated molecules or pathogen-derived products, and several cytokines such as interleukin (IL)-2, IL-12, IL-15, IL-18, IL-21, and interferon (IFN)-α/β (Vivier et al., 2008). Upon activation, NK cells proliferate, up-regulate effector molecules [e.g., perforin, granzymes, FasL, TNF-α, TNF-related apoptosis-inducing ligand (TRAIL)], kill target cells, and produce cytokines (e.g., IFN-γ and TNF-α) and chemokines (e.g., IL-8, MCP-1, MIP1-α, and RANTES) that influence the responses of other immune cells (Zamai et al., 1998; Wu and Lanier, 2003; Yokoyama et al., 2004; Vivier et al., 2008). Alterations in NK cell number or function (by an excess of inhibitory or activating signals) have been implicated in inflammation, carcinogenesis, and autoimmune diseases.

The TNF superfamily of ligands and receptors is critical for building innate and adaptive immune responses against foreign pathogens and cancer by regulating cell death and survival in cells under T cell or NK cell attack and by providing up- or down-regulatory effects on the killer lymphocytes themselves (Locksley et al., 2001; Ware, 2011). The TNF receptor (TNFR) family has two major sub-groups, death domain (DD)-containing receptors and TNFR-associated factor (TRAF) binding receptors. The DD receptors, such as Fas (CD95), TRAIL-R1, TRAIL-R2, and TNFR1, activate caspases via DD-containing adaptor proteins, leading to apoptosis (Chen and Goeddel, 2002; Ware, 2011). TRAF binding receptors, including CD27, CD40, OX40, HVEM, CD30 and 4-1BB (CD137; TNFRSF9), and GITR (CD357; TNFRS18), which are the subjects of this review, activate transcription factors [e.g., nuclear factor-kappa B (NF-κB)] and are associated with cellular activation, differentiation and survival. Various members of the TNF-TNFR superfamily are expressed by NK cells and can regulate NK cell responses (Watts, 2005; Bekiaris et al., 2009; Croft, 2009, 2010; Steinberg et al., 2011; Vujanovic, 2011; Rakhmilevich et al., 2012). New insights into the functions of 4-1BB and GITR distinguish these two TNFRs as NK regulatory receptors with dual functions, both activating and inhibitory. Their unexpected and intriguing properties in human NK cell-mediated anti-leukemia activity prompted this review and my discussion of therapeutic implications of modulation of 4-1BB-4-1BBL and GITR-GITRL interactions.

## Potential for manipulation of NK cells in tumor therapy

Research over the last decade has shown that NK cells are a promising tool for immunotherapy of cancers, in particular leukemia, that can be improved further by strategic modifications. Haploidentical stem cell transplantation (SCT) in acute myelogenous leukemia (AML), in which absence of KIR engagement on donor NK cells for the specific HLA molecules of the patients (termed KIR-HLA mismatch), produced powerful graft vs. leukemia (GVL) effects (Ruggeri et al., 2002). The NK cell KIR-HLA mismatches reduced risk for leukemia relapse without increased graft vs. host disease (GVHD). Patients with multiple myeloma who received haploidentical SCT also exhibited reduction in relapse rates, a benefit that may be NK cell mediated (Kroger et al., 2005). In addition, infusions of allogeneic NK cells are able to induce clinical remission in patients with AML (Curti et al., 2011; Nguyen et al., 2011; Miller et al., 2005). Infusions of NK cells have also improved the treatment of other cancers such as neuroblastomas, lymphomas, melanomas, and renal, ovarian, and breast cancers (Castriconi et al., 2007; Arai et al., 2008; Bachanova et al., 2010; Geller et al., 2011). Furthermore, Fc-FcγR interactions in NK cell mediated ADCC are important for the *in vivo* antitumor effects of the mAbs Rituximab (anti-CD20) for lymphomas, Herceptin (anti-Her2/neu) for breast cancers, Cetuximab (anti-EGFR) for head and neck cancer, and anti-GD2 for neuroblastomas (Clynes et al., 2000; Levy et al., 2011). Therefore, there is relevant *in vivo* therapeutic anti-tumor activity of NK cells that can be enhanced to make these cells even more important components of tumor control.

Attempts at increasing the susceptibility of a cancer cell to NK cell mediated lysis have focused on modulating the balance between inhibitory and activating NK cell receptor signals using blocking antibodies for inhibitory receptors, cytokines, and drugs. For example, blocking NK cell inhibitory receptors in hematopoietic SCT is being tested in clinical trials with a fully humanized anti-KIR monoclonal antibody (1-7F9) (Romagne et al., 2009; Sola et al., 2009; Benson et al., 2011, 2012; Vey et al., 2012). This monoclonal antibody recognizes KIR2D inhibitory receptors and blocks their interaction with the HLA-C molecules, leading to NK cell-mediated lysis of leukemia cells. However, as the recognition of MHC class I molecules by KIRs is crucial for developing NK cells to become functionally competence and discriminate “self” from “altered self” (NK cell education or “licensing”) (Kim et al., 2005; Anfossi et al., 2006), caution is warranted. IL-2 potently activates and induces NK cell anti-tumor activity, but systemic administration of this cytokine is associated with life-threatening toxicity (Fehniger et al., 2002). IL-15 or IL-21, which also enhance NK cell functions, might prove to be more effective than IL-2 [e.g., IL-2 induces activation-induced cell death (AICD) of cytotoxic lymphocytes and expands suppressive T regulatory cells (Tregs)] (Waldmann et al., 2001; Miller et al., 2005; Barao et al., 2011; Denman et al., 2012; Josefowicz et al., 2012) in ensuring *in vivo* persistence of transferred functional NK cells for long-term control of leukemia (and of other cancers). Also, certain drugs currently used in cancer therapy, such as Bortezomib, Lenalidomide, and Cyclosporin A have been shown to boost NK cell functions by induction NK cell-stimulatory ligands on tumor cells or cytokines (Poggi and Zocchi, 2005; Wang et al., 2007; Ames et al., 2009; Benson et al., 2011). Another approach could be the genetic modification of NK cells with tumor-specific chimeric antigen receptors (CARs) to amplify activating signals and induce specific killing of tumor cells. For example, coupling the activating domains of the 2B4 or 4-1BB to CD19 receptors and CD3ζ has shown to markedly enhance NK cell-mediated killing of CD19-positive leukemia cells (Imai et al., 2005; Altvater et al., 2009). Altogether, these achievements are encouraging observations to justify improvements in the *in vivo* NK cell retention that is needed to facilitate the many new approaches under consideration to manipulate NK cells against cancer.

## 4-1BB in NK cells

4-1BB is an inducible, co-stimulatory molecule expressed on activated CD4 and CD8 T cells. The majority of studies are centered on the use of 4-1BB agonistic antibodies or of 4-1BBL to increase the proliferation, function, and survival of T cells (Watts, 2005; Croft, 2009). Results are encouraging with systemic administration of 4-1BB agonistic antibodies in mouse models of T cell immunity toward tumors (Vinay and Kwon, 2012). On the basis of these results, 4-1BB anti-tumor properties are currently being tested in phase II clinical trials with a fully humanized 4-1BB agonistic mAb (BMS-663513) in patients with advanced solid malignancies and the antibody seems to have a favorable toxicity profile (Vinay and Kwon, 2012).

4-1BB is negligible on most resting NK cells and is induced on many of the NK cells upon activation with IL-2, IL-15, and CD16 triggering (Lin et al., 2008; Baessler et al., 2010). Initial studies showed that *in vitro* stimulation of mouse NK cells with 4-1BB agonistic antibodies or with cell lines expressing 4-1BBL induced NK cell proliferation and IFN-γ secretion, but without an increase of the spontaneous cytotoxicity that is the hallmark of NK cells (Melero et al., 1998; Wilcox et al., 2002). *In vivo* mouse and human xenograft tumor studies showed enhanced NK cell-mediated ADCC by 4-1BB triggering. Kohrt et al. reported that sequential administration of Rituximab (anti-CD20 mAb) followed by anti-4-1BB agonistic antibody treatment had potent anti-lymphoma activity in syngeneic mouse and human xenotransplanted lymphoma models (Kohrt et al., 2011). In addition, depletion of Tregs, which are known to suppress NK cells, could enhance anti-lymphoma activity (Houot et al., 2009). As well, agonistic antibody triggering of 4-1BB increased the response of mouse NK cells in mice bearing human Her2-overexpressing breast tumor cells and given Trastuzumab (anti-Her2 mAb) treatment (Kohrt et al., 2012). In addition, a recent study from Maniar et al. reported that human 4-1BBL-positive γδT cells induced robust human NK cell-mediated killing of tumors that are usually resistant to NK cytolysis (e.g., lymphomas, melanomas, breast, and colon tumors) through subsequent NKG2D recognition (Maniar et al., 2010). These studies indicate that in this mouse and human anti-tumor scenarios 4-1BB functioned as an activating NK cell receptor.

However, in sharp contrast to 4-1BB enhancements of the NK activities listed above, in the leukemia setting, 4-1BB-4-1BBL interactions can impair NK cell reactivity. Helmut Salih and coworkers found that 4-1BBL was highly expressed on many leukemias from patients with AML (23/65; 35%) and B cell chronic lymphocytic leukemia (B-CLL) (28/89; 32%) and that NK cells from these patients expressed 4-1BB (Baessler et al., 2010; Buechele et al., 2012a). Signaling via 4-1BB on allogeneic NK cells after binding to 4-1BBL-positive AML cells actually impaired NK cell functions (both cytotoxicity and IFN-γ), which were restored by blocking 4-1BB with specific antibodies (Baessler et al., 2010). A reduction in direct killing and Rituximab-induced NK cell ADCC against 4-1BBL-expressing B-CLL cells was also observed (Buechele et al., 2012a). These results are in remarkable contrast to the afore-mentioned 4-1BB-4-1BBL interactions that enhanced the reactivity of human T cells against AML cells (Houtenbos et al., 2007). Thus, 4-1BB functioned as an inhibitory NK cell receptor when NK cells were interacting with 4-1BBL-positive leukemia cells. Whether this direct inhibition can happen with other tumors that express 4-1BBL is presently unknown.

There is the potential for “double inhibition” of NK activity via cytokines as well as 4-1BBL from the leukemia cells. The ability to signal bidirectionally is a characteristic feature of many ligands of the TNF family (Eissner et al., 2004). Baessler et al. also reported that signals via 4-1BBL into leukemia cells stimulated the release of the immunosuppressive cytokines such as IL-10 and TNF-α (Baessler et al., 2010). IL-10 can suppress NK cell cytokine production (Mocellin et al., 2005) and TNF-α can induce NK cell apoptosis (Jewett et al., 1997). In fact, NK cell reactivity against 4-1BBL-positive leukemia cells could be restored by neutralization of IL-10 or TNF-α (with Infliximab) (Baessler et al., 2010). Overall, these findings indicate that 4-1BBL may actually enable leukemias to evade immune surveillance by inhibiting the anti-tumor activity of human NK cells through direct 4-1BB-4-1BBL interactions and by inducing release of immunosuppressive cytokines from the tumors.

How can the differences in outcome of 4-1BB triggering in mouse vs. human NK cells and NK vs. T cells be explained? The answer may lie in underlying differences in signaling molecules in the NK cells. Murine and human 4-1BB cytoplasmic domains recruit the adaptor proteins TRAF1 and 2, with 4-1BB having a higher affinity for TRAF2 (Arch and Thompson, 1998). The association of 4-1BB with TRAF2 results in activation of downstream signaling pathways, such as the NF-κB and mitogen-activated protein kinase (MAPK) pathways, including p38 and JNK activation, ultimately leading to T and NK cell activation, cytokine production, and cell survival (Wang et al., 2009). Human but not mouse 4-1BB can also recruit TRAF3 (Jang et al., 1998). Of note, TRAF3 forms heterotrimers with TRAF2 and inhibits NF-κB activation induced by TRAF2 following engagement of TNFRs such as CD40 and OX40 (Hauer et al., 2005; Hacker et al., 2011). Therefore, it is possible that in humans TRAF3 acts as a negative regulator of 4-1BB signaling leading to NK cell inhibition by NF-κB inactivation. Moreover, human and mouse 4-1BB intracellular domains differ by small sequences of amino acids that might bind regulatory molecules such as phosphatases (Marvel and Walzer, 2010). In fact, one of these motifs, present only in human 4-1BB resembles an immuno-tyrosine inhibitory motif (Marvel and Walzer, 2010). Reciprocally, mouse but not human 4-1BB contains the sequence Cys-X-Cys-Pro which mediates the binding of co-receptors such as CD4 and CD8 to the protein tyrosine kinase, p56^lck^ (Kim et al., 1993); this kinase is essential for the optimal responses of T cells to antigen. For perspective, p56^lck^ is expressed by NK cells (Salcedo et al., 1993). The opposite outcome of 4-1BB triggering in NK and CD8 T cells in humans can compounded by differential expression of inhibitory receptors (e.g., KIRs and NKG2A/B) and by heterodimerization between TNFR members (i.e., OX40 and 4-1BB in T cells (Ma et al., 2005). In summary, it is only possible now to speculate on the contributions to differences in 4-1BB effects on mouse vs. human NK cells and NK vs. T cells. Additional studies are required and important because the murine models may mislead human clinical strategies.

## GITR in NK cells

Studies in mice have shown that GITR activation, either by anti-GITR antibodies or by its ligand GITRL increases TCR-induced T cell proliferation and cytokine production and rescues T cells from anti-CD3-induced apoptosis (Watts, 2005). Consistent with this co-stimulatory role of GITR for T cells, additional studies have shown that modulation of the GITR-GITRL interaction with agonistic antibodies can be an effective immunotherapy for tumors that are immunogenic for T cells (Placke et al., 2010; Nocentini et al., 2012). Therefore, an agonistic human anti-GITR mAb (TRX-518, Tolerx^R^), which blocks the interaction of GITR with its ligand and also co-stimulates T cells and enhances the cytotoxicity of NK cells, is currently under clinical investigation for the treatment of melanoma [(Schaer et al., 2010) and clinicaltrials.gov].

Although there is a consensus for the role of GITR in mouse T cells, its role in NK cells is controversial. There is little information about GITR with mouse NK cells. GITR engagement can be activating or inhibitory to human NK cells. GITR is constitutively expressed at low levels on NK cells and up-regulated after activation with IL-2 and IL-15 (Baltz et al., 2007; Liu et al., 2008). Hanabuchi et al. found that activated plasmacytoid DCs (pDCs) expressing GITRL enhanced NK cell cytotoxicity and IFN-γ production via GITR-GITRL interactions (Hanabuchi et al., 2006). Using GITRL-expressing transfected target cells they also showed that signaling by GITR promoted NK cell lysis in synergy with IL-2, IFN-α, or anti-NKG2D antibodies. Therefore, GITRL can work synergistically with other stimulants to promote NK cell activation. In contrast, Baltz et al. reported that engagement of GITR on activated NK cells by soluble (s) GITRL (from cultures of tumor cells) or GITRL-Ig fusion protein or GITRL expressed on the tumor target cells inhibited NK cell functions (cytotoxicity and IFN-γ) (Baltz et al., 2007, 2008). A marked reduction of the transcription factors c-Rel and RebB was observed, indicating that GITR negatively modulates NF-κB activity (Baltz et al., 2007, 2008). Neutralization of sGITRL with GITR-Ig fusion proteins partially restored the levels of RelB and increased NF-κB activity and NK cell capacities (Baltz et al., 2008). Along with this study, Liu et al. reported that GITR signaling with anti-GITR mAbs concurrently inhibited NK cell proliferation as a result of blocked phosphorylation of Stat5 and Akt downstream signaling proteins. GITR ligation also resulted in reduced NK cell cytokine production (i.e., IFN-γ, IL-1, and IL-6) and increased NK cell apoptosis (e.g., low expression of the anti-apoptotic proteins Bcl-XL and Bad) (Liu et al., 2008). In aggregate, these studies indicate that GITR has both positive and negative regulatory functions for NK cells *in vitro*.

The intracellular signaling of GITR involves multiple TRAFs as do 4-1BB. In fact, GITR signals downstream through a trimer consisting of a single TRAF5 and two TRAF2 molecules (Schaer et al., 2012). Available data indicate that whereas TRAF1, 2, 4, and 5 activate NF-κB, TRAF3 antagonizes the effects of TRAF2 in NF-κB activation (Placke et al., 2010; Hacker et al., 2011). A precedent for differential regulation mediated by different adaptor molecules has been described for the NK cell receptor 2B4 in mice and men (Lanier, 2005); 2B4 engagement can be stimulatory or inhibitory depending on the signaling molecules that are associated (Roncagalli et al., 2005). Also, in the context of receptors and signal transduction, structural oligomeric differences between murine and human GITRL (e.g., primarily dimers in mouse vs. trimers and superclusters in human) (Nocentini et al., 2012) may contribute to different responses by the receptor-bearing cells. It is clear that more information is needed to define the mechanisms by which GITR affects NK cell activity.

Like 4-1BBL, GITRL is capable of transducing signals into the GITRL-expressing tumor cells (reverse signaling) after engagement. Batlz et al. observed that GITRL signaling down-regulated the expression of CD40 and CD54/ICAM-1, and induced tumor cell production of immunosuppressive TGF-β (Baltz et al., 2007). Since CD40 and C54 mediate activation and adhesion of immune cells, their lower expression on tumor cells may diminish NK cell binding to tumors and cytotoxicity. TGF-β suppresses NK cell functions (Ghiringhelli et al., 2005; Barao et al., 2006). Thus, GITRL expression seems to affect the interaction of tumor cells with the immune system by influencing tumor cell immunogenicity and creating an immunosuppressive cytokine microenvironment.

The Salih group reported that, besides 4-1BBL, high expression of GITRL was observed on leukemia cells in many patients with AML (34/60; 57%) and CLL (47/60; 78%), but 4-1BB and GITR were absent on healthy CD34^+^ hematopoietic stem cells (Baessler et al., 2009; Buechele et al., 2012b). The leukemic patients had high levels of sGITRL in their sera. These observations suggest that GITRL and 4-1BBL may be biomarkers of increased malignancy. In addition, GITR was expressed on the NK cells of these patients. Primary AMLs expressing GITRL inhibited the function (direct killing and IFN-γ) of allogeneic NK cells (Baessler et al., 2009). Direct killing or Rituximab-induced ADCC of B-CLL cells mediated by autologous NK cells were also reduced by GITR triggering through cell-cell contact or sGITRL (Buechele et al., 2012b). GITRL signaling into various leukemia cells induced the production of TNF-α, IL-6, and IL-8, or IL-10 (Baessler et al., 2009; Buechele et al., 2012b) that not only enhanced proliferation and survival of leukemia cells but also could suppress the immune system. Blocking GITR on NK cells with anti-GITR mAbs or neutralizing sGITRL, IL-10 or TNF-α has the potential to restore NK cell functions (direct killing, ADCC, and IFN-γ). A recent report from Placke et al. also indicated that GITRL-expressing platelet-coated tumor cells (termed GITRL pseudoexpression) were able to inhibit NK cell function (Placke et al., 2012). The described results indicate that GITR-GITRL interactions contribute to evasion of leukemias from NK immunosurveillance.

## Concluding remarks

The development of cancer is dependent on the interaction of tumor cells with the immune system, a reciprocal process that influences whether transformed cells are eliminated or progress to a life-threatening disease. As NK cells emerge as therapeutic agents for cancer treatment, it is important to define whether a given cancer is susceptible to NK cell responses and to develop customized therapeutic approaches that involve reduction of NK cell suppression and/or stimulation of NK cell reactivity. Here I reviewed what is currently known for the roles of the TNFRs 4-1BB and GITR in NK, focusing on human anti-leukemia activity. 4-1BB and GITR can apparently mediate both inhibitory and activating signals on NK cells. With leukemias, there is an opportunity to induce a sufficient NK cell response to prevent relapses in patients after haploidentical HSCT which unfortunately occur in 11–48% of the patients, depending on the clinical considerations (Hu et al., 2011). Many patients with leukemia have high levels of s4-1BBL and sGITRL in their sera; therefore, it will be important to determine whether these levels influence the final outcome of their disease and to determine whether neutralization of the s4-1BBL and/or sGITRL using R-Ig fusion proteins can enhance NK cell-mediated tumor control (Figure 1B). The use of blocking anti-4-1BB or anti-GITR mAbs to disrupt interactions is another possible approach to prevent NK cell inhibition. However, the occurrence of reverse signaling (from the R-Ig fusion proteins) and/or potential simultaneous attenuation of T cell responses (by blocking mAbs) after these therapeutic interventions have to be taken into account when considering the benefits of promoting NK anti-leukemia activity. Of note, Tregs highly express GITR, but suppression of human Tregs, in contrast to mouse Tregs, does not seem to be inhibited by this receptor (Placke et al., 2010). Further studies will better elucidate the differential effects of 4-1BBL and GITRL signaling in mice and men and in different immune situations (i.e., T cells vs. NK cells and during tumor cell killing or during prior interactions with other immune cells). Adjustments of the weight(s) in the balance of activation vs. inhibition signals of NK cells have implications for safety and efficacy of approaches to exploit 4-1BB and GITR to treat cancer.

### Conflict of interest statement

The author declares that the research was conducted in the absence of any commercial or financial relationships that could be construed as a potential conflict of interest.
